# The Impact of Human Salivary Amylase Gene Copy Number and Starch on Oral Biofilms

**DOI:** 10.3390/microorganisms13020461

**Published:** 2025-02-19

**Authors:** Dorothy K. Superdock, Lynn M. Johnson, Jennifer Ren, Alizeh Khan, Megan Eno, Shuai Man, Angela C. Poole

**Affiliations:** 1 Division of Nutritional Sciences, Cornell University, Ithaca, NY 14853, USA; 2 Cornell Statistical Consulting Unit, Cornell University, Ithaca, NY 14853, USA

**Keywords:** *AMY1*, amylase, oral microbiome, oral microbiota, periodontitis, caries, cavities, starch, biofilm

## Abstract

The copy number (CN) variant *AMY1* encodes the salivary amylase enzyme which promotes starch digestion. Although this gene has been associated with dental caries and periodontal disease susceptibility, the impact of the interaction between *AMY1* CN and starch on oral biofilms is unclear. We explored how oral microbiota communities shaped by *AMY1* CN respond to starch by employing an in vitro model of biofilm formation. We cultured biofilms using saliva samples from 31 donors with a range of *AMY1* CNs (between 2 and 20 copies) and self-reported gum disease states; we used media with and without starch. Many of the most prevalent genera in saliva were also prevalent in the derived biofilms. The presence of starch in the media was associated with lower biofilm alpha diversity. We found a significant interaction between *AMY1* CN and the media carbohydrate content that influenced the proportions of *Atopobium* and *Veillonella*. Members of these genera have been associated with dental caries and periodontitis. These findings suggest that the effects of carbohydrates on oral microbiome composition depend on *AMY1* CN and that human oral bacteria evolved in response to expansion of this host gene locus.

## 1. Introduction

The human salivary amylase enzyme is encoded by the gene *AMY1* which initiates the digestion of rapidly digestible starches in the mouth. Evidence suggests that rapidly digestible starches increase the risk of dental caries, whereas slowly digestible starches (e.g., whole grains) may protect against periodontitis [1]. *AMY1* varies in gene copy number (CN) from 2 to 20 among individuals [2]. *AMY1* CN is correlated with salivary amylase concentration as assessed by western blot as well as salivary amylase enzyme activity (SAA) as measured in kinetic assays [3]. Furthermore, several environmental factors, including stress and circadian rhythms, influence SAA [3,4]. *AMY1* duplications are believed to have undergone positive selection following the Neolithic Revolution—the transition to an agricultural lifestyle that resulted in increased starch availability [2,5,6]. At that time, more copies of a gene involved in starch digestion may have provided a fitness advantage by increasing the calories extracted from starch. Yet, some of the concomitant phenotypic consequences may not be beneficial. For example, *AMY1* CN has been associated with oral pathologies, such as periodontitis and dental caries.

Dental caries is one of the most prevalent non-communicable diseases worldwide [7,8]. The development of dental caries involves the fermentation of carbohydrates (mainly sugars) by the bacteria in dental biofilms that produce acid and cause demineralization of the tooth’s surface [9]. In addition, the breakdown of rapidly digestible starchy foods can cause an acute decrease in the pH of dental plaque [10]. Initially, this demineralization can be reversed by removing the biofilm with saliva or tooth brushing, especially in the presence of fluoride; however, if the process is not interrupted, cavitation occurs. Early studies implicated *Streptococcus mutans* as causing dental caries [11], and subsequent epidemiological studies have identified additional genera (*Actinomyces*, *Atopobium*, *Bifidobacterium*, *Scardovia*, and *Veillonella*) as being associated with dental caries progression [12].

Salivary amylase has been associated with dental caries, but the nature of the association is in dispute. Studies of adult populations in Lithuania and Mexico reported that *AMY1* CN is positively correlated with the number of dental caries [13,14]. However, researchers who performed a study comparing *AMY1* knockout to wild-type mice concluded that amylase has a protective effect against dental caries [15]. This finding is supported by studies reporting a higher SAA in caries-free children compared to children with caries [16,17].

Salivary amylase has also been associated with periodontal disease, or gum disease, a chronic inflammatory disease of the gums that is prevalent worldwide [18,19,20,21,22]. Periodontal disease begins with plaque buildup, which can progress to inflammation and infection (gingivitis), gum recession, and periodontitis, resulting in severe deterioration and eventual tooth and bone loss [23]. Several studies have reported higher salivary amylase concentrations in individuals with periodontal disease than controls (people without periodontal disease or people who have recovered). This indicates that more amylase is being produced in response to the disease state to mediate a protective effect [24,25,26,27]. However, these reports did not consider the *AMY1* CN of the participants. Previous studies have shown that periodontitis is characterized by an oral microbiota that differs from that of individuals without gum disease [28], including an increased presence of several microbes, such as *Porphyromonas gingivalis* and *Porphyromonas endodontalis* [29,30,31].

In a study assessing *AMY1* CN and oral microbiome composition, we previously found that individuals with a high *AMY1* CN (≥9) have salivary microbiomes containing higher proportions of *Porphyromonas endodontalis* compared to individuals with a low *AMY1* CN (≤5) [32]. *Porphyromonas endodontalis* is found in higher abundance in periodontal disease sites [33]. Therefore, we hypothesized that a high *AMY1* CN could be a risk factor for periodontal disease because of the pathogenic potential of the associated oral microbiota.

We used saliva samples from human donors with a range in *AMY1* CN to seed cultures with oral bacteria, adapting a previously published in vitro system [34]. Each saliva sample was used to culture biofilms in triplicate in two media: subSHI and subSHI containing 0.1% starch. We predicted that inoculating cultures with saliva from individuals with more copies of *AMY1* would enhance the growth of periodontitis-associated pathogens within in vitro biofilms. We also predicted that starch would interact with *AMY1* CN to affect biofilm community composition due to differences in the capabilities of the bacterial species correlated with *AMY1* CN to degrade starch versus sugar as a carbon source. Identifying the taxa involved will inform precision medicine strategies to prevent oral diseases.

## 2. Materials and Methods

### 2.1. Saliva Collection

We collected human samples and data as part of a Cornell University Institutional Review Board-approved protocol #1902008575, approved on 18 March 2019. All participants provided written informed consent. These samples were collected during a study in which we were analyzing oral and gut microbiomes. The inclusion and exclusion criteria for this study are listed in Table S1. We asked individuals to refrain from brushing their teeth for at least six hours and eating or drinking for at least 30 min before sample collection. In a survey, participants self-reported their gum disease status as one of four categories: periodontitis, gingivitis, gum recession, or none. We obtained 5–10 mL of saliva from each donor, who donated their sample through passive drooling for the collection of unstimulated saliva into a 50 mL sterile conical tube. Saliva samples were immediately placed at 4 °C, aliquoted within three hours either into empty microcentrifuge tubes or microcentrifuge tubes containing a final volume of 30% glycerol to serve as a cryoprotectant, and stored at −80 °C until further use. We used the samples stored in glycerol for culturing experiments only and performed the rest of the assays on the samples stored without glycerol.

### 2.2. AMY1 Copy Number Determination by Quantitative PCR and Droplet Digital PCR

We used the QIAamp 96 DNA Blood Kit, QIAamp Blood Mini Kit, and the QIAamp Investigator Kit (Qiagen, Germantown, MD, USA, cat # 51161, 51104, 56504) to extract genomic DNA from saliva samples. We performed quantitative PCR (qPCR) to amplify *AMY1* and *EIF2B2* (our reference gene, with CN = 2) with primers, as described in Poole et al. [32]. For each gene, each 10 μL qPCR reaction consisted of 1 μL genomic DNA at 5 ng, 0.5 μL of each forward and reverse primer at 10 μM, 3 μL of PCR grade H_2_O, and 5 μL iTaq™ Universal SYBR^®^ Green Supermix (Bio-Rad Laboratories, Hercules, CA, USA, cat # 1725122). Using a Roche LightCycler^®^ 480 Real-Time PCR Instrument, the qPCR conditions were as follows: initial denaturation at 95 °C for 5 min, 40 cycles at 95 °C for 10 s, and 60 °C for 30 s. On each reaction plate, we developed a standard curve using genomic DNA NA12286 (Coriell Institute; *AMY1* CN = 2), including negative controls containing PCR grade water only, and including the following genomic DNA with previously estimated *AMY1* CNs as positive controls: NA18972, NA12873, NA10472, NA12890, NA10852, NA12043, NA11992, NA12414, NA12340, NA06994, NA12342, NA12286, NA18522, and NA19138 (Coriell Institute for Medical Research, Camden, NJ, USA). All reactions were performed in quadruplicate and results were averaged for technical replicates with a coefficient of variation of less than 0.05. At least two qPCR runs were performed for all saliva samples and the median value of all qPCR results was used as the final qPCR *AMY1* CN value. For digital droplet PCR (ddPCR), genomic DNA was digested with HaeIII (New England Biolabs, Ipswich, MA, USA, cat # R0108S). The DNA was diluted to 15 ng/µL before ddPCR was performed in duplicate reactions using Life Technologies TaqMan™ Copy Number Assay Id Hs07226361_cn for *AMY1* (Thermo Fisher Scientific, Waltham, MA, USA) and TaqMan Copy Number Reference Assay Hs06006763_cn for *AP3B1* to normalize for total DNA on a QX100 Droplet Digital PCR System (Bio-Rad Laboratories, Hercules, CA, USA). We averaged the median of all qPCR results and the two ddPCR results to determine the final *AMY1* CN used in our analyses. For two of the donors, we did not obtain qPCR results, and thus only the two ddPCR results were averaged for use in the analyses.

### 2.3. Salivary Amylase Activity

We measured the SAA for each saliva sample using the Salimetrics Salivary Alpha-Amylase Enzymatic Kit (SALIMETRICS, Carlsbad, CA, USA, cat # 1-1902). The assay was performed for each sample with two to three technical replicates and results were averaged to determine the final SAA for each donor. We followed the manufacturer’s protocol except we used 300 µL amylase substrate per reaction instead of 320 µL. As the SAA is known to be influenced by numerous external factors, saliva samples were chosen based on the correlation between *AMY1* CN and SAA.

### 2.4. Media

The SHI medium is a complex growth medium that was first developed by Tian et al. to support supragingival bacterial communities and the growth of diverse biofilms inoculated with a diverse microbial community [35]. The subSHI-v1 medium was later developed by Lamont et al. [34] to better support microbes associated with a disease-state subgingival microbial community. In this paper, we refer to this latter media as “subSHI” media. In brief, this media contained proteose peptone (10 g/L), trypticase peptone (5 g/L), yeast extract (5 g/L), KCl (2.5 g/L), hemin (5 mg/L), vitamin K (1 mg/L), urea (0.06 g/L), L-arginine (0.174 g/L), *N*-acetylmuramic acid (0.01 g/L), mucin (2.5 g/L), sucrose (0.1%), sheep’s blood (5%), and fetal bovine serum (10%), combined in ultrapure Milli-Q water (MILLI-Q Water System from Millipore, Burlington, MA, USA). For our modified subSHI media containing starch, we added starch to the subSHI media to obtain a final volume of 0.1% starch.

### 2.5. Biofilm Cultures

We adopted the biofilm culturing protocol previously described by Lamont et al. [34], except we inoculated cultures using saliva samples instead of subgingival plaque. Using each saliva sample, we precoated wells of a 48-well non-tissue-culture treated plate to create a salivary pellicle to facilitate biofilm adhesion. Saliva samples were centrifuged at 2600× *g* for 10 min to remove any debris and the resulting supernatant was added to one well. The plate was tilted to coat the wells completely. The remaining supernatant was then transferred to the next well to be coated until four wells for that individual saliva sample were coated. The remaining supernatant was recollected. We incubated plates at 37 °C for one hour then treated them with ultraviolet light for an additional hour to dry and sterilize the saliva pellicle. After the pellicles were dried, we added 495 µL of media to the three wells that were inoculated and 500 µL to the fourth well (a pellicle-negative control). We used 5 µL of each saliva sample to inoculate our biofilm cultures in media with and without starch (Figure S1). We then incubated plates under anaerobic conditions (Coy Anaerobic Chamber) for 48 h at 37 °C. After incubation, we harvested biofilms from wells. We scraped the bottom of the wells to collect adherent cells and used PBS buffer as needed to facilitate the dislodging of biofilms. Biofilms were stored at −80 °C until DNA extraction.

### 2.6. Microbial DNA Extraction and 16S rRNA Sequencing

We thawed biofilms and their corresponding saliva inocula and centrifuged all samples at 14,000× *g* for five minutes. We then used the DNeasy PowerSoil Pro Kit (Qiagen, cat #47014) following the manufacturer’s protocol to perform DNA extraction on each sample pellet and used a final elution volume of 50 µL. We included extraction blanks with each DNA extraction batch. We determined the DNA concentration using the Quant-iT™ PicoGreen™ dsDNA Assay Kit (Invitrogen, Carlsbad, CA, USA, cat #P11496). All reagents, including TE, PCR water, and primers were tested and confirmed to be contaminant-free for up to 25 PCR cycles. To prepare libraries of the 16S rRNA v4 region for sequencing, we performed PCR amplification in duplicate 50 µL reactions, each containing 1–500 ng DNA, 0.5 µL each of 515F and 806R primers [36], and 25 µL Classic ++ Hot Start Taq DNA polymerase Master Mix (Neta Scientific, Marlton, NJ, USA, cat #TB-31-5011-1000R). We followed PCR cycling conditions as previously described except with 25 cycles. We purified amplicons using Mag-Bind^®^ TotalPure NGS (Omega Bio-Tek, Inc., Norcross, GA, USA, cat #M1378-01) and pooled 100 ng of each sample. Sequencing was performed using Illumina MiSeq (Illumina, Inc., San Diego, CA, USA) 2 × 250 paired-end sequencing.

### 2.7. Bioinformatics and Statistical Analysis

We used QIIME 2 for microbiome sequence data analysis [37]. We used q2-demux to demultiplex and quality filter raw sequences and q2-dada2 (DADA2) to denoise and fragment-insertion SEPP to construct a phylogenetic tree using the Greengenes 13_8 99% identity reference tree [38,39]. We used q2-diversity to calculate diversity metrics after samples were subsampled without replacement to 12,789 sequences per sample to retain the sample with the lowest sequencing count. We used q2-feature-classifier classify-sklearn naïve Bayes classifier against the Greengenes 13_8 99% OTUs reference sequences for taxonomic assignments [40,41].

All statistical analyses were performed in RStudio using R versions 4.2.1–4.4.1 [42]. We generated PCoA plots for biofilms and saliva samples using an unweighted UniFrac distance matrix containing the distances between each donor’s saliva sample and its associated biofilms. For our PERMANOVA analyses, we used the adonis2 function in the vegan package version 2.6.8 to fit the model adonis2(distance.matrix ~ participant + sample_type, data, by = “terms”) [43]. After subsetting the matrix to only include biofilm cultures, we fit the model adonis2(distance.matrix ~ media + participant, data, by = “terms”).

We compared the alpha diversity of the saliva samples to that of the biofilm samples with and without starch. There were 31 saliva samples and 179 biofilm samples. We used R packages lme4, lmerTest, and emmeans. For the biofilm samples, we used the mean of the alpha diversity values of the technical replicates for each media to balance the groups. We used linear mixed models with Faith’s phylogenetic diversity (Faith’s PD) and Pielou’s evenness as the response variables, which were log-transformed. We used the model: log (alpha_diversity) ~ sample_type + (1|donor), where alpha_diversity is Faith’s PD or Pielou’s evenness; sample_type is saliva, media with starch, or media without starch; and donor is a random effect, which was included because each donor contributed each of the three sample types.

The analyses of the biofilm samples included saliva donor age, media type, and gum disease status as covariates. There were 119 biofilm samples derived from donors with no reported gum disease, 18 from donors with gingivitis, 24 from donors with gum recession, and 18 from donors with periodontitis included in this analysis, of which 92 were grown in the subSHI media and 87 in the subSHI + 0.1% starch media. For the alpha diversity analysis, we used the following linear mixed model: log (Faith’s PD of biofilms) ~ gum_disease + donor_age + media + (1|donor) + (1|donor:media) to measure the effect of media (starch versus non-starch) and gum disease, adjusting for age and using donor as a random effect. Because there were three technical replicates per donor per media type, we included a random effect of media nested in donor, (1|donor:media). The inclusion of the interaction term *AMY1* CN × media or SAA × media did not significantly influence the model and was removed. Post hoc pairwise comparisons between gum_disease types and media types were performed using Tukey’s HSD method to adjust for multiple comparisons. For taxonomic abundance differences, we used the following model: Genus ~ gum_disease + donor_age + AMY1CN + media + AMY1CN × media + (1|donor) + (1|donor:media).

## 3. Results

### 3.1. Characteristics of Saliva Sample Donors

**Figure 1:**
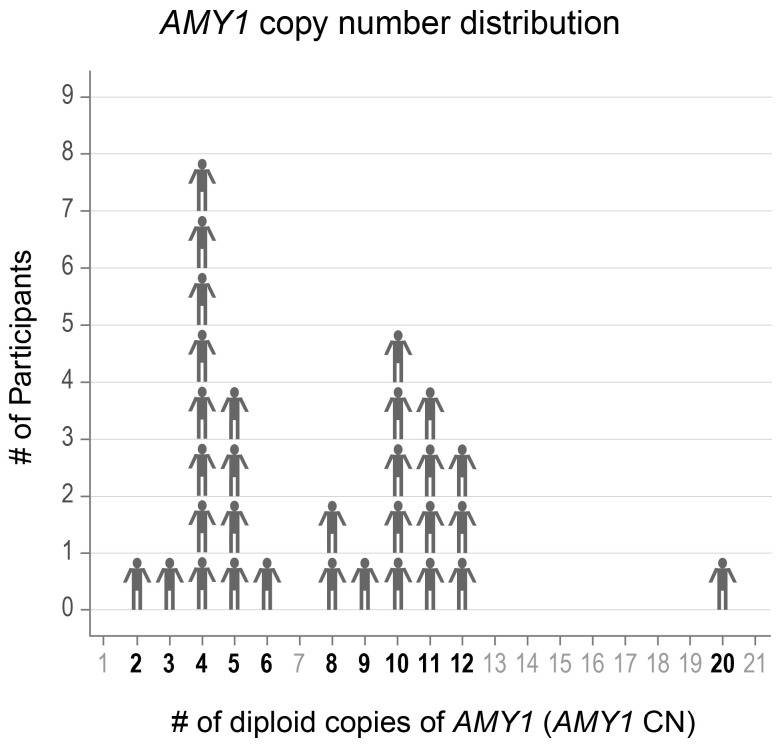
The *AMY1* copy number (CN) of participants ranged from 2–20. The CNs of the participants included in the study are shown in bold font.

We selected 31 adults aged 19–57 years old with a range in *AMY1* CN from whom we had collected multiple saliva samples (Figure 1). Participants indicated whether they had ever been diagnosed with gingivitis, gum recession, periodontitis, or none of these. We also recorded the age of the participants at the time of saliva donation, as age is known to influence saliva microbiota composition [44]. Donor characteristics are presented in Table 1.

### 3.2. Biofilm Microbial Communities Differ from Saliva Microbial Communities and Maintain Donor Identity

We visualized differences in overall microbiome composition between saliva inocula and biofilm samples using principal coordinates analysis (PCoA) plots of unweighted UniFrac distances. Here, we used one saliva sample and its corresponding biofilm cultures in each media type per donor. We performed a permutational multivariate analysis of variance (PERMANOVA) to assess the contribution of specific factors to the observed variation in microbial community composition. Participant identity explained 29% of the observed variation in microbial community composition (*p* = 0.001), and sample type explained 32% (*p* = 0.001) (Figure S2). Evaluating biofilms alone, participant identity explained 55% of the observed variation (*p* = 0.001), and media type explained 3.2% (*p* = 0.001) (Figure S3).

**Figure 2:**
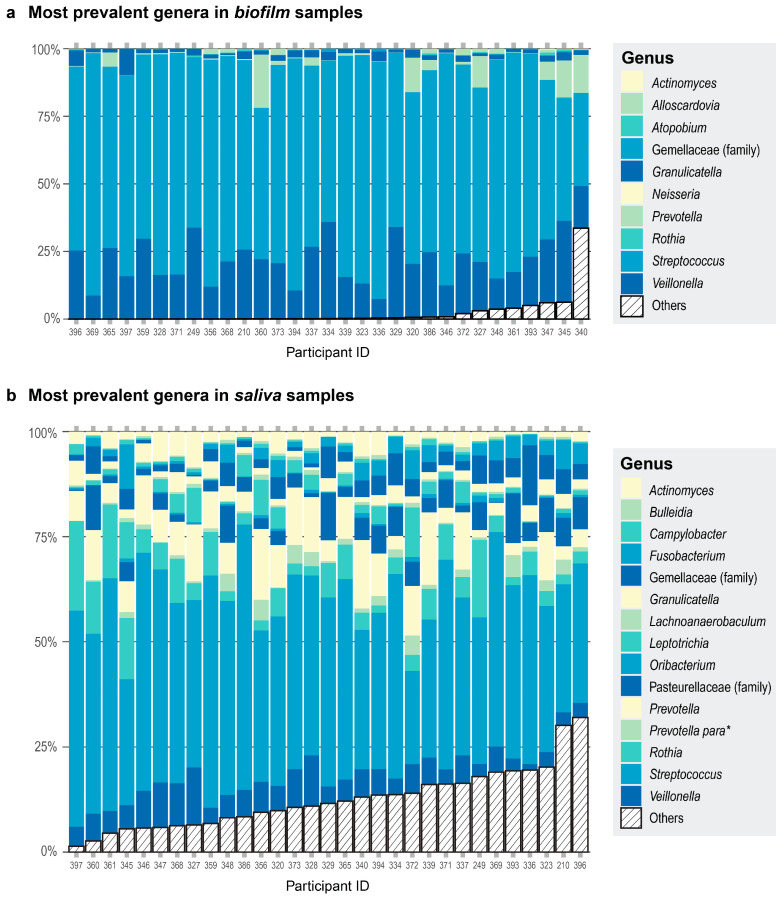
Each participant is represented by a column. Participant ID is on the *x*-axis. The color assignments differ between panels, and some are used more than once within each panel. (**a**) Proportions of the top 10 most prevalent genera in the biofilm samples are shown in different colors and any other genera present are collapsed in the category Others (striped bar). The top three genera were present in the same number of samples. (**b**) Proportions of the top 15 most prevalent genera in the saliva samples are shown in different colors and any other genera present are collapsed in the category Others (striped bar). All 15 genera were present in all the donors. * *Prevotella para* is an abbreviation for the genus *Prevotella* currently assigned to the family Paraprevotellaceae in the Greengenes database.

We also observed differences in community composition when we compared the most prevalent genera in saliva versus biofilm samples. All 31 of the saliva samples had 15 genera in common (Figure 2a). The 10 most prevalent genera in the biofilm samples comprised 98% of the read counts from the biofilm samples (Figure 2b). When comparing the most prevalent genera in the saliva and biofilm samples, there were seven genera in common—*Streptococcus*, *Veillonella*, *Granulicatella*, Gemellaceae (genus unclassified), *Prevotella*, *Rothia*, and *Actinomyces*. Alpha diversity was greater in the saliva samples than in the biofilm samples cultured in starch or non-starch media when assessed using Faith’s phylogenetic diversity (Faith’s PD; F (df1 = 2, df2 = 59) = 354.6, *p* < 0.001) and Pielou’s evenness (F (df1 = 2, df2 = 59) = 34.48, *p* < 0.001). These results are expected because some of the microbes are lost during sample collection, storage, and freeze-thawing, and culturing conditions cannot exactly recapitulate the environment of the native habitat of the microbes. Nevertheless, donor identity was conserved—the biofilm communities derived from a donor were more similar to each other than to the biofilm communities from another donor (Figure S2).

### 3.3. Phylogenetic Diversity of Biofilm Cultures Is Predicted by Self-Reported Gum Disease Status and Media

**Figure 3:**
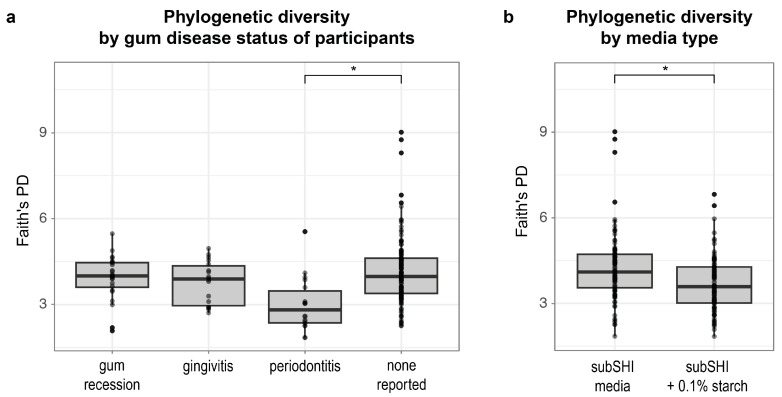
The alpha diversity of biofilms is associated with self-reported gum disease status and starch (* *p* < 0.001). (**a**) Faith’s phylogenetic diversity (PD) is significantly lower in biofilms cultured from donors with periodontitis compared to biofilms cultured from donors with no reported gum disease. (**b**) Faith’s PD is significantly lower in biofilms cultured in starch-added media.

Next, we identified factors that predict differences in phylogenetic diversity within biofilms. Using a linear mixed model, we found that alpha diversity (measured by Faith’s PD) was significantly different between levels of gum disease status (F (df1 = 3, df2 = 27) = 5.1; *p* = 0.0065), and media (F (df1 = 1, df2 = 28) = 23.05; *p* < 0.001). Specifically, Faith’s PD was significantly lower in biofilms cultured from saliva from donors with periodontitis compared to saliva from donors with no form of gum disease reported (*p* < 0.001) (Figure 3a), adjusting for saliva donor age and media type (with or without starch). We also found that Faith’s PD was significantly lower in starch-supplemented media compared to media without starch (*p* < 0.001) (Figure 3b), adjusting for saliva donor age and gum disease status. Neither AMY1 CN nor SAA alone (nor their interactions with media type) predicted Faith’s PD of biofilms in our dataset (*p* > 0.15).

### 3.4. The Effect of Starch Supplementation on the Relative Abundances of Veillonella and Atopobium in Biofilms Is Modified by AMY1 CN

**Figure 4:**
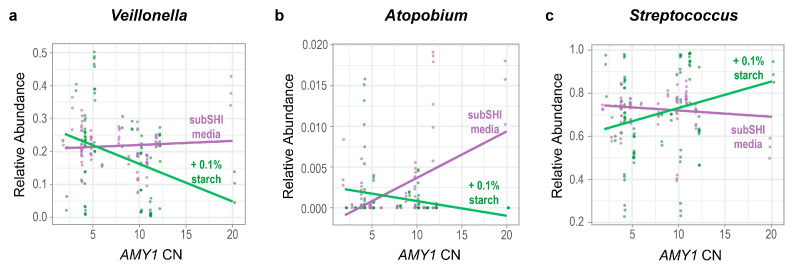
The relative abundances of *Veillonella* and *Atopobium* are influenced by an interaction between *AMY1* CN and media. For high *AMY1* CN, the relative abundances of (**a**) *Veillonella* (*p* = 0.038) and (**b**) *Atopobium* (*p* = 0.001) are lower in starch-supplemented media. (**c**) There is a trend for the opposite relationship in *Streptococcus*; for high *AMY1* CN, the relative abundance of *Streptococcus* (*p* = 0.078) is higher in starch-supplemented media.

We tested whether the relative abundances of genera were influenced by an interaction between *AMY1* CN and starch. Ordered from most to least prevalent, the top ten most prevalent taxa in our biofilm cultures were *Streptococcus*, *Veillonella*, *Granulicatella*, *Gemellaceae* (genus unclassified), *Prevotella*, *Neisseria*, *Rothia*, *Atopobium*, *Actinomyces*, and *Alloscardovia*, with the top three genera being equally prevalent (Figure 2b). Because these 10 genera constituted 98% of the sequence read counts in the biofilm dataset, we used a linear mixed model for each to test whether there was a significant difference in the relative abundance of each genus depending on both the *AMY1* CN of the donor and whether there was starch in the media. We also included gum disease status and saliva donor age as covariates in the models. We found that the relative abundances of *Veillonella* and *Atopobium* were significantly influenced by an interaction between *AMY1* CN and media (F (df1 = 1, df2 = 27) = 4.73, *p* = 0.038 for *Veillonella* and F (df1 = 1, df2 = 27) = 12.8, *p* = 0.001 for *Atopobium*) (Figure 4a,b). As *AMY1* CN increases, the relative abundances of both these genera decreases in starch-supplemented media. By contrast, the relative abundances of both genera increase in media without starch as *AMY1* CN increases. We also observed a trend in the opposite direction for differences in the relative abundance of *Streptococcus* depending upon the presence of starch in the media (Figure 4c; F (df1 = 1, df2 = 27) = 3.37; *p* = 0.078). As *AMY1* CN increases, the relative abundance of *Streptococcus* is higher in media with starch than without but, as *AMY1* CN increases, the relative abundance decreases in media without starch.

We also observed significant effects of age, media, gum disease, and *AMY1* CN on several genera (Table 2). *Streptococcus*, *Prevotella*, and *Atopobium* had age as a significant predictor (*p* < 0.05). Relative abundances of *Granulicatella*, Gemellaceae, *Atopobium*, and *Alloscardovia* were predicted by the presence/absence of starch in the media (*p* < 0.05). Finally, the relative abundance of *Atopobium* increased with *AMY1* CN (*p* = 0.023), and the relative abundance of *Granulicatella* was significantly higher in periodontitis biofilms than in healthy biofilms (*p* = 0.025).

## 4. Discussion

Our findings demonstrate the tractability of this in vitro model for assessing the impact of host attributes and dietary substrate on microbial community composition. Although cultured biofilm communities differed from salivary communities, many of the most prevalent genera in saliva were detected in the biofilms. We found that donor identity was a strong predictor of cultured biofilm community composition. Thus, the individuality of samples from the same donor was maintained, a phenomenon that has been observed across human body sites [45].

We evaluated the influence of *AMY1* in the presence and absence of starch on salivary microbial communities. For a high *AMY1* CN, *Veillonella* and *Atopobium* have lower relative abundances if the media is supplemented with starch—suggesting that species within these genera have lower fitness than other bacteria when starch is an available substrate. *AMY1* CN is positively correlated with salivary amylase activity. Since greater salivary amylase activity promotes a greater breakdown of starch into simpler carbohydrates, the oral microbiota in a high *AMY1* CN mouth may be adapted to use simple sugars as a carbon source. The preferred carbohydrate source of most *Veillonella* spp. is lactate, a breakdown product of sugar metabolism. Veillonellae cannot metabolize glucose, fructose, and disaccharides, e.g., sucrose, the sugar in the media used in our study [46]. Veillonellae depend on other species, including streptococci, that metabolize sugars and are known to co-aggregate with early and late colonizers of oral biofilms. A metagenomic analysis of nine type strains revealed that some strains of *Veillonella atypica* possess CAZymes [47], suggesting that there may be unknown carbohydrate utilization in some strains of this species.

There are conflicting reports regarding the pathogenicity of different *Veillonella* spp., but they are frequently found in higher abundance in dental caries [48] and could be an early risk factor for the dysbiotic state that leads to dental caries. One study found that an unidentified growth molecule produced by *Veillonella parvula* promotes the growth of *P. gingivalis* and leads to periodontal bone loss in mice [49]. *P. gingivalis*, a keystone species in periodontitis, can be difficult to culture in biofilms outside of its natural environment and requires a high degree of specialization including, but not limited to, media optimization, coaggregation with other species, and a high inoculum load of purified culture [50,51].

We also observed that, for a high *AMY1* CN, *Atopobium* has a lower relative abundance if the media is supplemented with starch. *Atopobium* has been associated with dental caries [52,53,54,55], and some members of the genus can degrade complex carbohydrates [56]. One potential explanation of why *Atopobium* spp. from a high versus a low *AMY1* CN display different growth in the media could be that different species or strains within this genus have greater fitness depending upon the available substrate: starch or sugars. The Carbohydrate-Active enZYmes (CAZy) Database has gene sequences from carbohydrate-active enzymes (CAZymes) found in species of *Atopobium* [57], so the species detected in our analyses may be exhibiting substrate preferences depending upon the presence/absence of different CAZymes. Alternatively, changes in *Atopobium* abundance could result from supportive or competitive interactions with other microbes influenced by *AMY1* CN.

We observed a trend for *Streptococcus* to have higher relative abundance if the media is supplemented with starch. Streptococci are known to metabolize carbohydrates generating acids as breakdown products [58], and several *Streptococcus* spp. are known to bind to salivary amylase [59]. *Streptococcus* species, such as *S. mutans* and *S. gordonii*, have been shown to mediate oral biofilm formation [60,61,62]. However, *S. sanguinis*, also present in biofilms, is a human oral commensal associated with health [63], and has been shown to inhibit the cariogenicity of *S. mutans* [64]. Hence, the finding that starch supplementation favors the growth of *Streptococcus* in high *AMY1* individuals suggests that a higher *AMY1* CN may contribute to biofilm composition in individuals with diets high in rapidly digestible starch. However, we could not comprehensively distinguish between commensal and pathogenic species in these experiments using 16S rRNA gene sequence data.

Additionally, we observed that the diversity of microbial communities within biofilms was significantly different, depending on the gum disease status of the saliva donor. Biofilms cultured from the saliva of donors with periodontitis had significantly lower alpha diversity than those with no gum disease reported. Another study that measured the alpha diversity of oral samples (saliva and subgingival plaque) collected from individuals with and without periodontitis reported that alpha diversity is higher in the disease state, which is the opposite of what we observed in our biofilm cultures [65]. However, another study performed metagenomic profiling of subgingival plaque from individuals with varying progression of periodontitis and reported a loss of oral diversity as disease severity increased [66]. We also found that supplementing the media with starch decreased alpha diversity, which could be due to the preferential growth of microbes capable of using starch or the breakdown products of starch as a substrate.

One limitation of our study is that in our sample size of 31 individuals who donated saliva samples, the 11 donors who had a form of gum disease self-reported their condition. Since gingivitis is reversible, we do not know the status at the time of saliva donation. Furthermore, gum recession and periodontitis are not mutually exclusive but are overlapping conditions. Future work should include a larger number of samples from healthy donors and from donors with each form of gum disease confirmed at the time of sample donation. This may allow researchers to characterize the timing of the progression of community changes and how they correlate with disease severity; this could, in turn, suggest tailored probiotic therapies appropriate at different time points of intervention. Future studies should also include shotgun metagenomics analysis for higher resolution of the taxonomy to potentially reveal strains of bacteria promoting biofilm formation and benign strains that can outcompete them. An additional limitation is that we used saliva to seed the cultures as opposed to dental plaque, but one advantage of using saliva is that we had enough inoculum to create technical replicates in different types of media. Finally, this in vitro system may not fully represent the complex dynamics of the oral environment. We did not vary salivary amylase activity in the cultures. We isolated the response of the bacterial communities, which had adapted to the milieu of the host’s *AMY1* CN, to the carbohydrate substrates provided in the media.

The results from our study have implications for the advancement of precision dentistry [67]. As indicated by the burgeoning field of salivaomics, there is great potential for saliva to be used as a biomarker for oral conditions [68]. Since *AMY1* CN and the presence of starch are predictive of changes in community composition, genotyping people at this locus could be informative for oral hygiene practices. Depending upon their *AMY1* CN, people may be advised to minimize consumption of rapidly digestible starch or to brush their teeth afterward if they are at higher risk for dental caries or periodontitis.

## 5. Conclusions

Our findings support the premise that the effects of carbohydrate intake on oral health are mediated by bacteria associated with *AMY1* CN. The consumption of different forms of carbohydrates, e.g., sugars versus rapidly digestible starches, could promote the growth of different species or strains of bacteria that influence the pathogenicity of oral biofilms on the gums and teeth. Additionally, the expansion of this gene locus may have resulted in the adaptation of the host’s oral bacteria to promote their growth on the carbohydrate sources that remain following digestion by host enzymes. This study provides evidence of an important interplay between diet, genetics, and oral microbiota, offering new insights into the impact of evolution on oral health.

## Figures and Tables

**Table 1:** Characteristics of the donors who provided the saliva samples used in the analyses.

Total n	31
Age in years (Mean ± SD)	19–57 (29.5 ± 9)
Sex	
Female	25 (81%)
Male	6 (19%)
Gum disease status (self-reported)	
Gingivitis	3 (9.7%)
Gum recession	4 (12.9%)
Periodontitis	4 (12.9%)
None	20 (64.5%)

**Table 2:** The *p*-values for the terms included in the linear models assessing the interaction between *AMY1* copy number (CN) and media type (starch versus non-starch). Significant *p*-values are in bold font.

Prevalence	Taxonomy	Gum Disease	Age	*AMY1* CN	Media	*AMY1* CN × Media
1	*Streptococcus*	0.77	**0.038**	0.62	**0.079**	**0.078**
1	*Veillonella*	0.42	0.38	0.21	0.17	**0.038**
1	*Granulicatella*	**0.025**	0.89	0.27	**<0.0001**	0.97
4	Gemellaceae	0.35	0.73	0.84	**0.0097**	0.28
5	*Prevotella*	0.52	**0.0030**	0.93	0.10	0.65
6	*Neisseria*	0.45	0.71	0.13	0.16	0.43
7	*Rothia*	0.43	0.48	0.33	0.49	0.81
8	*Atopobium*	0.18	**0.0015**	**0.023**	**0.016**	**0.0013**
9	*Actinomyces*	0.38	0.022	0.63	0.99	0.49
10	*Alloscardovia*	0.89	0.52	0.60	**0.032**	0.29

## Data Availability

Upon publication of the manuscript, the raw fastq sequences generated from the 16S rRNA amplicon sequencing will be available in the National Center for Biotechnology Information Sequence Read Archive via the project number PRJNA1195917.

## Supplementary Materials

The following supporting information can be downloaded at: https://www.mdpi.com/article/10.3390/microorganisms13020461/s1, Figure S1: Experimental design; Figure S2: PCoA of saliva and biofilms; Figure S3: PCoA of biofilms; Table S1: Inclusion and exclusion criteria.
