# Supplementary material for: The Impact of Human Salivary Amylase Gene Copy Number and Starch on Oral Biofilms
Source: Microorganisms. 2025 Feb 19;13(2):461. doi: 10.3390/microorganisms13020461 (PMC11858026; doi:10.3390/microorganisms13020461)
Supplement: Supplementary file 1 [file microorganisms-13-00461-s001.zip › microorganisms-3400389-supplementary.pdf]

Inclusion criteria	Exclusion criteria
Aged 18 years or above	History of diabetes, prediabetes or impaired glucose tolerance
Willing to have height, weight, waist, and hip circumference measured	An existing, UNTREATED, thyroid condition
Willing to provide 4 saliva samples and 12 stool samples over ~7 weeks of the study duration	Use of systemic antibiotics (intravenous injection, intramuscular, or oral) within the last 6 months
Stable weight (±5 pounds) for the last three months	An acute disease at the time of enrollment (Acute disease is defined as the presence of a moderate or severe illness with or without fever).
	A chronic, clinically significant (unresolved, requiring ongoing medical management or medication) pulmonary, cardiovascular, gastrointestinal, hepatic, or renal functional abnormality
	A history of active uncontrolled gastrointestinal disorders or diseases including<br>A. Inflammatory bowel disease (IBD)<br>i.) Ulcerative colitis (mild-moderate-severe)<br>ii.) Crohn's disease (mild- moderate-severe)<br>B. Indeterminate colitis<br>C. Irritable bowel syndrome (IBS) (moderate-severe)<br>D. Persistent, infectious gastroenteritis, colitis or gastritis, persistent or chronic diarrhea of unknown etiology<br>E. Clostridium difficile infection (recurrent)<br>F. Chronic constipation
	Bariatric surgery
	An unstable dietary history as defined by major changes in diet during the previous month. For example, elimination or significantly increased intake of a major food group in the diet.
	A recent history of chronic alcohol consumption defined as more than 5 drinks (or servings) of alcohol per day.
	Female participants: Pregnancy and/or lactation

Table S1. Inclusion and exclusion criteria.
This table provides the inclusion and exclusion criteria for study participants.

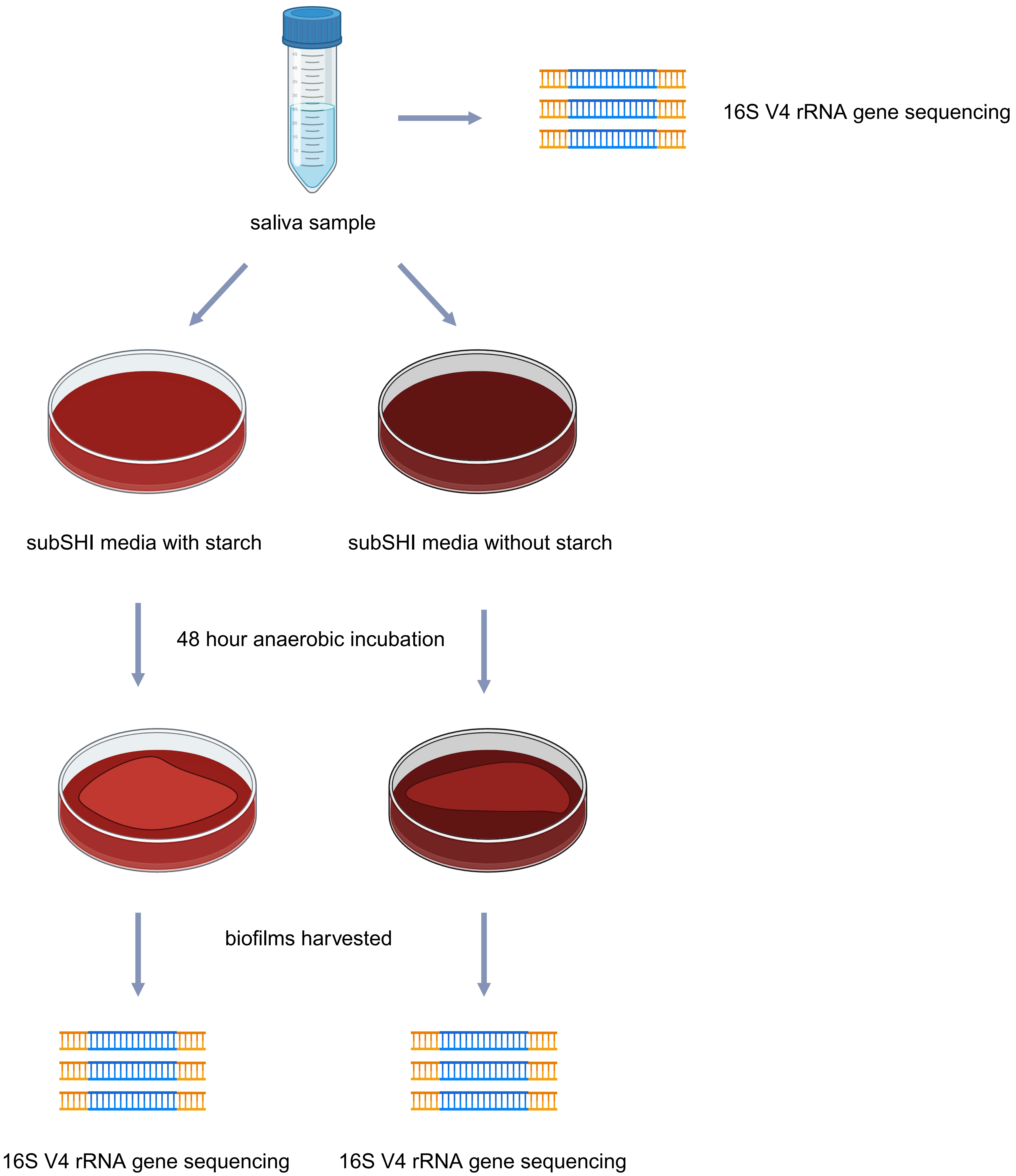

Figure S1. The experimental design is shown here. Prior to the steps shown, sample is pipetted into the well and dried to provide a matrix for the biofilm to adhere. The plates are then exposed to ultraviolet light to destroy the DNA. Afterwards, a fresh aliquot of sample and media are added to the well as shown above. Created in BioRender. Superdock, D. (2025) https://BioRender.com/h23o738

Figure S2. Unweighted UniFrac for Saliva Inoculum and Biofilm Cultures

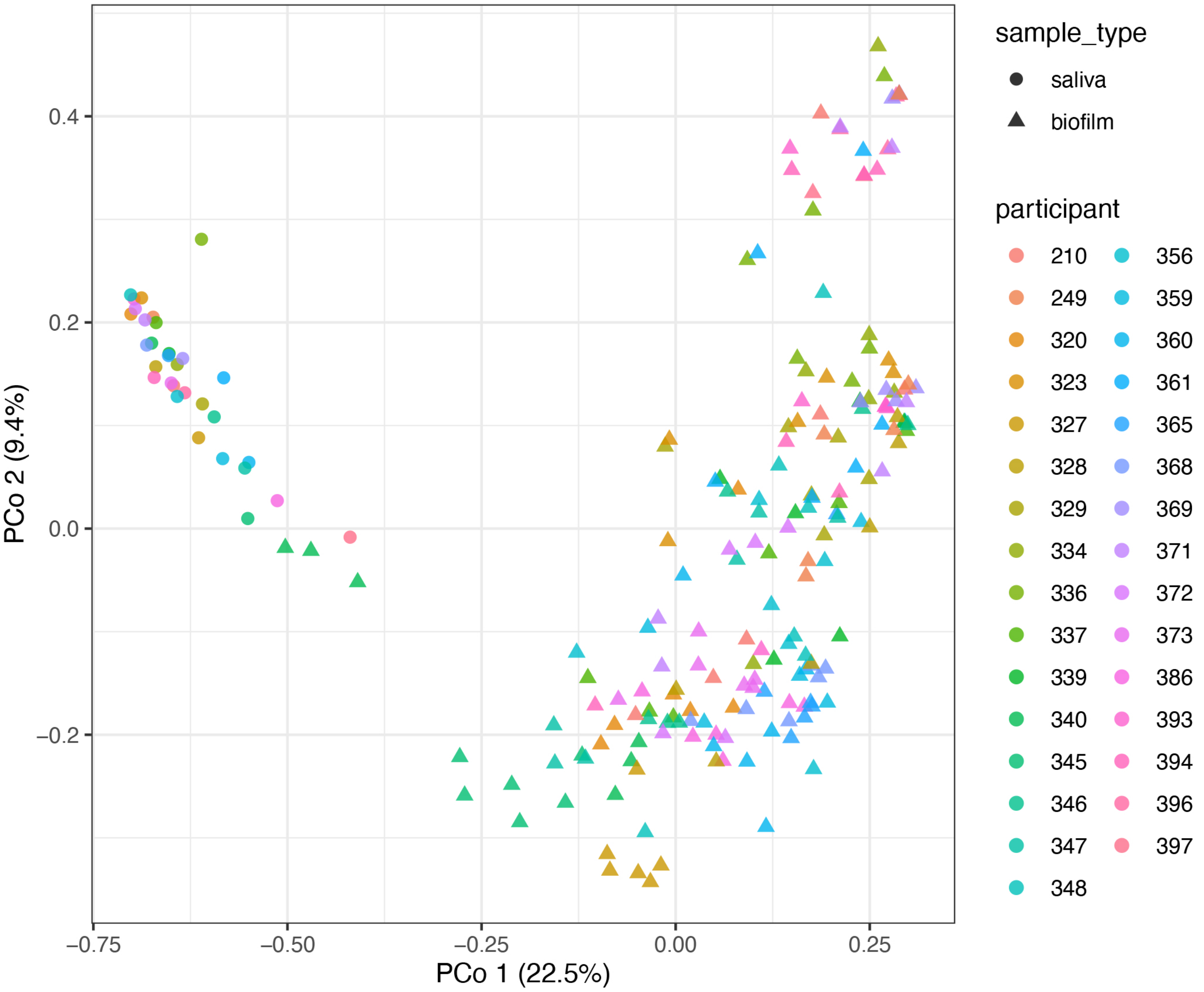

Figure S2. This principal coordinates analysis plot was created using unweighted UniFrac. Sample type is indicated by shape, and donor identity is indicated by color..

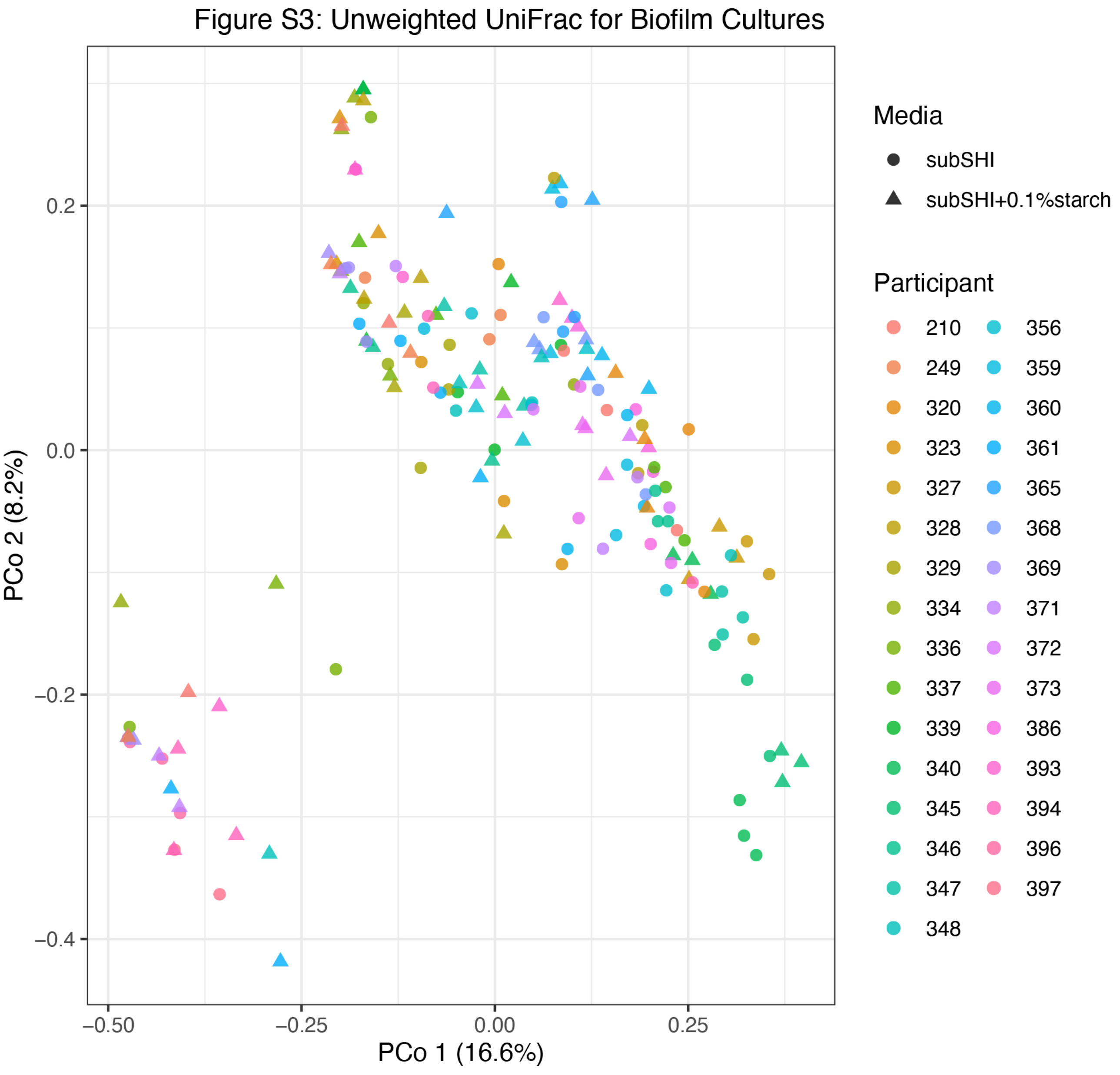

Figure S3. This principal coordinates plot was created using unweighted UniFrac. Media type is indicated by shape, and donor identity is indicated by color.
